# Acknowledgement to Reviewers of *Biomolecules* in 2019

**DOI:** 10.3390/biom10010145

**Published:** 2020-01-16

**Authors:** Biomolecules Editorial Office

**Affiliations:** MDPI AG, St. Alban-Anlage 66, 4052 Basel, Switzerland

The editorial team greatly appreciates the reviewers who have dedicated their considerable time and expertise to the journal’s rigorous editorial process over the past 12 months, regardless of whether the papers are finally published or not. In 2019, a total of 941 papers were published in the journal, with a median time to first decision of 17.5 days and a median time from submission to publication of 38 days. The editors would like to express their sincere gratitude to the following reviewers for their generous contribution in 2019:
A.C., CefalasKrzysztof, ZienkiewiczAamir, AhmadKshitij, KhatriAaron, EngelhartKuan Rong, ChanAbdel, AouacheriaKuniaki, OgasawaraAbdelhakim, BouyahyaKuo Hao, LeeAbheepsa, MishraKurt, GrüngreiffAbhijeet A., BakreKustiariyah, TarmanAbhijit, RangnekarKwan Ho, TangAbraham, Wall MedranoKyle, BurghardtAchim, KochKyoungtae, KimAchraf, NoureddineKyu, LimAdam, EkielskiKyunghee, ByunAdam, MatkowskiKyung-Soo, ChunAdam, LiwoKyung-su, KimAdebowale, OgunjirinL. Omur, DemirezerAdela, PenesovaLachlan, MitchellAdela, PinteaLahiru, JayakodyAditi, BanerjeeLarisa, PoluektovaAdolfo, SaiardiLarisa, KordyukovaAdrian Gabriel, TorresLarry, OldhamAdriana E., MieleLarryn, PetersonAdriano, Costa De CamargoLars, MalmströmAdriano, MendesLars, GilleAdrienne, GormanLars Ove, BrandenburgAdrover, MiquelLászló, JicsinszkyAfshin, MosahebiLászló, HazaiÁgnes, TantosLaura, BlairAgnieszka, FiszerLaura, GiamperiAgnieszka, AdamskaLaura, CatenacciAgnieszka, Paradowska-GoryckaLaura, StraussAgnieszka, SliwinskaLaura, SabatinoAgnieszka, GornowiczLaura M., BrutscherAgostino, MiglioreLaurence, LeherteAhmed, HusseinLaurence, RisAhmed, BakillahLaurence, Meslet-CladiereAhmed, ArslanLaurent, ChiarelliAhmed O., MoslehLaurent, MetzingerAhmed Q., HaddadLauri, PolariAhmet, KertmenLaurie, ShornickAimy, SebastianLavinia, VlaiaAjeet, KaushikLee Lee, WongAkinori, KuzuyaLefkothea, PapadopoulouAkio, MorinobuLei, WanAkira, YamashitaLenka, LuhováAkira, SugawaraLenka, KubicovaAlain, Favre-RéguillonLeo R., QuinlanAlain, WuethrichLeonard E., GerberAlamelu, SundaresanLeonardo V M, De AssisAlan, StewartLeticia, González-MayaAlan, CastleLetícia, Scherer KoesterAlanna, GreenLetizia, GiampietroAlba, SilipoLi, BianAlbert, Selva-O'CallaghanLi, DiAlberto, MartínezLi (Licky), ZhuAlejandra, CovarrubiasLiana, SalantaAlejandro, Samhan-AriasLianbo, LiAlejandro, RomeroLiang, LiangAlejandro, MadridLih-Geeng, ChenAleksandar Ž., KostićLilin, GeAleksander, CzogallaLiming, BianAleksandr, KakinenLina, JinAleksandr A., SoldatovLindsey, KennedyAleksandra, PawlakLindsey, Van HauteAlena, OpattovaLionel, VerdoucqAlenka, CopicLiping, ChenAleš, GregorcLirong, MengAlessandra, PolliceLisa, CrawfordAlessandra, DurazzoLisa, MarinelliAlessandra, StacchiottiLisheng, CaiAlessandro, TerrinoniLiu, ZhaopengAlessandro, MalobertiLiu, FilipeAlessandro, BorgiaLiwei, LuAlessandro, MartoranaLizabeth, AllisonAlessio, PapiniLong, Nguyen PhuocAlessio, AccardiLong-Liu, LinAlessio, GambinoLoredana, MoroAlex, DickensLorenza, DíazAlexander, MacKerellLoukas, KanetisAlexander, ShpakovLuba, TchertanovAlexander, BerezinLubos, CipakAlexander, V IvanovLuca, RosciniAlexander, LorenzLuca, TianoAlexander, BuchbergerLuca, LiberaleAlexander, BarteltLuca, LaraiaAlexander, LjubimovLucas Danilo, DiasAlexander, BerestetskiyLuchang, ZhuAlexander, LeitnerLucia, BorrielloAlexander, TimoshenkoLucia, MendozaAlexander H, KirschLucia, PanzellaAlexander M, JonesLucia, MontenegroAlexander TH, WuLucía, García-OrtegaAlexandr Alexandrovich, KolobovLucía, Inglada-PérezAlexandra, WormitLuciana, ScottiAlexandra, FidalgoLuciano, PirolaAlexandre, EscargueilLucie, BednárováAlexei, LiceaLucilla, IacuminAlexey, KhodjakovLucio, MerliniAlexey M., PetrovLucjusz, ZaprutkoAlfonso, CornejoLucyna, KirczukAlfonso, MangoniLudmilla, Morozova-RocheAlfonso, Garcia-SosaLudovic, BaraultAlfredo, BudillonLudovic, JeanAlfredo, VellidoLuigi, De BellisAli, ElgamelLuigi, MilellaAli, MovahediLuigi, ZeccaAli, SoleimanzadehLuigi, MessoriAlice, VilelaLuis, BriebaAlicia, RoqueLuis, RojoAlicja, Kuban-JankowskaLuis, SantosAlicja, BanasiakLuis, Fernandez-TorresAlicja, WegrzynLuis Carlos, MartínezAlin, CiobicaLuís Filipe, RibeiroAlison, HarrillLuis Guillermo, Hernández-MontielÁlvaro Efrén, Domínguez RebolledoLuiz Henrique, Mendes Da SilvaAmandeep, KaurLuiza, Breitenfeld GranadeiroAmedeo, LonardoLuiza, FrankAmelie, Heuer-JungemannLukas, HawinkelsAmit, KetkarLukas, PacalAmit, KumarLukáš, NajdekrAmosy, M'KomaLukáš, ŽídekAmr, El-DemerdashLukasz, KozubowskiAmy, KigerŁukasz, OpalińskiAn, GhyselsŁukasz, WojtylaAna, DuarteŁukasz, UramAna, Mendes-FerreiraŁukasz, SzeleszczukAna, BorotaŁukasz, SobottaAna, MornarLu-Kwang, JuAna A., Feregrino-PérezLyndsay V., RhodesAna Beatriz, Furlanetto PachecoM, NasserAna Čipak, Čipak GašparovićM. Gregor, MadejAna Maria, TeixeiraM. Hasan, MohajeriAna Mendes, LealMa, NamboodiriAnanya, PaulMa. Xenia G., IlaganAnastasia V, ShindyapinaMaciej, SzaleniecAnca, MacoveiMaciej, SałagaAnca, StanaMaciej, WłodarczykAnca, ToiuMaciej, MasłykAnders, AxelssonMaciej, JaskiewiczAndras, PerlMaco, ConsumiAndre, PalmerMadhuvanthi, VijayanAndré, MeloMafalda, PintoAndrea, BeccioliniMagdalena, WięcekAndrea, GerbinoMagdalena, Wójciak-KosiorAndrea, FusoMagdalena, Paplińska-GorycaAndrea, MastinuMagdalena, ZukAndrea, RagusaMahboubeh, PishnamaziAndrea, SalzanoMahmoud A.O., DawoodAndrea, SorannoMahmud K A, FoyezAndrea, LapucciMaja, LeitgebAndreas, KriegMaja, GrabackaAndreas, LeunigMaja, MatulićAndreas, Von KnethenMakoto, YamaguchiAndreas, HeylandMakoto, EndoAndreas, TzakosMalgorzata, CzyzAndreas, MeyerMalgorzata, Latos-BrozioAndreas, WreeMalgorzata, KowalskaAndreas S., KalogirouMałgorzata, Maciążek-JurczykAndreea, SoareMałgorzata, DżuganAndrei, SurguchovMałgorzata, GniewoszAndrei, SmertenkoMałgorzata, Lasik-KurdyśAndrei, PotapovMałgorzata, GrabarczykAndrei, AnghelMamdouh, El-ShishtawyAndrei A., PotaninManabu, MoriyaAndreina, SchoeberleinManish, ShahAndrés González, SantanaManish, TripathiAndrew, TsotinisManoj, KhadkaAndrew David, GreenhalghManuel, González-HidalgoAndrey, IvanovManuel, Baño-PoloAndrzej, KloczkowskiManuela, ViolaAndrzej, KasperskiMao Lun, WengAndrzej, ParzonkoMarat R, TalipovAndrzej, RapakMarc, SuretteAndrzej, GalatMarc, YesteAndrzej, TeisseyreMarçal, GallemíAngel Luis, García-VillalónMarcel, BermudezAngela, CelettiMarcela De Sá Barreto, Da CunhaAngelita, GambutiMarcello, LocatelliAngelo, NacciMarcelo, FerreiraAngelo, GismondiMarcin, LukaszewiczAngelo, LavanoMarcin, NowickiAngelo, MaffeiMarcio, TieraAngelo M, CarellaMárcio, RodriguesAngus, CameronMarcio Vinicius, Bertacine DiasAnikó, BorbásMarco, FiliceAnikó, PósaMarco, NardiniAnil, SinghMarco, GiovineAnimesh, AgarwalMarco, JoseAnita, MikolajczykMarco, SchiavoneAnita, UmerskaMarco, ConfalonieriAnita, PinarMarco, AbbatangeloAnita, ScipioniMarco, SalerAnna, PawełczykMarco A., Loza-MejíaAnna, KawiakMarco A., RamosAnna, MichalskaMarcus, GroettrupAnna, LewinskaMarcus, ScottiAnna, ChojnackaMarcus, StåhlmanAnna, BlendaMarcus O. W., GrimmAnna, CalarcoMarek, KieliszekAnna, KotMarek, SanakAnna, BiernasiukMarek, OrłowskiAnna, SparatoreMargaret, SlavinAnna, Di SalleMargaret Dah-tsyr, ChangAnna, AjdukMargherita, EufemiAnna, Villar-PiquéMargherita, MaioliAnna, JakubczykMaria, PedreroAnna, AntsiferovaMaria, CubellisAnna Maria, GrimaldiMaria, DimarogonaAnna Maria, PosadinoMaria, WrobelAnna Renata, DembskaMaria, VivoAnna Yu, SpivakMaria, AlexandriAnna-Karin, Borg-KarlsonMaria, ZamaraewaAnnalisa, SantucciMaria, MonsalveAnnarita, FalangaMaria, GazouliAnne, VanetMaria, GaczynskaAnne, BaronMaria, CarraraAnne Karine, LagendijkMaria, CaeiroAnne-Sophie, PetitotMaria, De MieriAnnika, Kengelbach-WeigandMaria, João FerreiraAnsgar B., SiemerMaria Cristina, CaroleoAnshu, GuptaMaria Angeles, Tormo-MasAnthi, KarnaouriMaria Antonietta, CasadeiAnthony, ByrneMaria Carla, MarcotullioAntoaneta, PopovaMaria Carmen, PuertasAntoine, MaruaniMaria Cristina, De RosaAntoine, TalyMaria Cristina, VinciAntonella, PasqualoneMaria Cristina, KenneyAntonia, TerpouMaría D., CouceAntonino, NatalelloMaria Das Gracas Lins, BrandãoAntonino, BelfioreMaria De Lourdes, Ribeiro De Souza Da CunhaAntonio, PigaMaria De Lurdes, DinisAntonio, CicchellaMaria Del Mar Ortega-villaizan, RomoAntonio, Magán-FernándezMaria Do Socorro, RufinoAntonio, Di StefanoMaria E.M., AraujoAntonio, GiordanoMaria Eugenia, ArizaAntonio, GalindoMaria Francesca, SantollaAntonio Juan, PazosMaria Giulia, LionettoAntony, Packiam-DayalanMaria Graziella, De MontisAnu, KauppinenMaría Jesús, OrtegaAnuj, KumarMaria José, Pérez AlvarezApostolos, ZaravinosMaría L., LópezArash, NavranMaria Lucia Carneiro, VieiraArash, KhakiMaria Manuel B., MarquesArchie, ReyesMaria Manuela, GasparArduino A, MangoniMaria Marjorette, PenaAri M., HietalaMaria Rita, MarquesAria, BaniahmadMaria Rosa, Pérez-GregorioAriane, RozzaMaría Soledad, PratsAriane, MüllerMarian, BresticArianna, RicciMariana, Saucedo-GarcíaArijit, NathMariana, BrozmanováAristidis S., VeskoukisMariana Carmen, ChifirucArkadiusz, OrzechowskiMariana S., CastroArmando E., González-StuartMariane, LutzArnaud, ChatonnetMariangela, MarrelliArrigo, CiceroMarianne, De VilliersArthur L., DeVriesMariateresa, VolpicellaArti V., ShindeMariateresa, RussoArtur, GryszkinMarie, BillaudArtur, BeberokMarie Lue, AntonyArtur, GóraMariela, BollatiArturo, MugaMarie-Lise, JobinArturo J., CardounelMarika, KarikoskiArulselvan, PalanisamyMariko, SaitoArun, PanduranganMarina, CostaAruna, BitraMarina, PozzoliniArvi, RaukMarina, MapelliÅslög, DahlMario, OlleroAthanasios, KimbarisMário Antônio Alves, Da CunhaAthina, GeronikakiMario D., GalignianaAtsuko, Kamijo-IkemoriMario Milco, D’EliosAtsushi, HosuiMariusz, MojzychAtsushi, SakaiMariusz, MamińskiAttila, HunyadiMark, MoehleAtul, SrivastavaMark, KindyAudrey A., VasauskasMark, RosenfeldAurel, Popa-WagnerMark C., HoustonAurélia E., LewisMark S., ButlerAutar K., MattooMarko, NovinecAvinash Chandra, PandeyMarkus, LaubeAvril A. B., RobertsonMarla, BerryAxel, MithoeferMarshall, WilliamsAyami, MatsushimaMarta, De ZottiB. I., IorgaMarta, Plonska-BrzezinskaB. Montgomery, PettittMarta E., Alarcón-RiquelmeBa Thuy Linh, NguyenMartha, AssimakopoulouBabken, AsatryanMartha Patricia, GallegosBachir, BenarbaMartin, SmieskoBakhtiyor, RasulevMartin, MichaelisBakier, SlawomirMartin, SchlesingerBalaji, NagarajanMartin, BakhtBanafsheh, MehrazmaMartin, KrátkýBang-An, WangMartin, Michaelis, Prof.Bangjun, ZhouMartin R, GillBaolin, GuoMartin S., StaegeBaoyin, ZhaoMartine, AbboudBarak, RotblatMartiniano, BelloBarbara, TomaselloMary, CloningerBarbara, WisniowskaMary C., Farach-CarsonBarbara, BlackwellMary J., GuttieriBarbara, MolesiniMary J.C., HendrixBárbara, AngelMasaaki, KonishiBárbara, PortoMasaaki, KanemoriBarry, CheungMasafumi, TanakaBart, HoogenboomMasahiro, NakajimaBart, VanhaesebroeckMasakazu, AtobeBart, RuttemanMasaki, OkumuraBartosz, AdamczykMasami, NakazawaBassam, AbomoelakMasamichi, YamanakaBastian, FrommMasamichi, IkeguchiBauke W., DijkstraMasanao, YokohiraBeata, PajakMasanobu, NannoBeata, JastrzebskaMasao, KishidaBeata, StaniszMasao, NakagawaBeata, Morak-MłodawskaMasaru, HoshinoBeata, LemliMasashi, IdogawaBeata, StrzemieckaMasashi, KitazawaBeata, SkibskaMasato, YanoBeatriz Salinas, Salinas RodríguezMasayasu, KuwaharaBen C. L., ChanMasfique, MehediBenedikt, LinderMassimiliano, BianchiBenoît, MenandMasumi, SuzuiBenoît, Van AkenMáté, VargaBernadette, JulierMateusz, PsurskiBernardo Gonçalves, AlmeidaMateusz, LabudaBernat, CrosasMateusz, MaciejczykBernhard, BiersackMatheus, FroeyenBert, De RybelMathias, WinterhalterBertrand, LiagreMathieu, Van Der JagtBeth, LeeMathieu, HautefeuilleBetsy, LeverettMatteo, ScampicchioBhagwan Sahebrao, BatuleMatteo T., DegiacomiBharath Kumar, VelmuruganMatthew, TaylorBhesh, SharmaMatthew, RätsepBhupesh, SinglaMatthew, McIntoshBianca, MoldovanMatthew, SpragueBijun, ZengMatthias, ThalmannBilon, KhambuMátyás, MinkBing, ZhuMaura, Téllez-TéllezBirgit, LeitingerMaurice B., HallettBirsen, CakirMauricio, MoraisBithiah Grace, JaganathanMaurizio, PesceBjarke, FollinMax, CryleBlas, Lotina-HennsenMaxim Y., KhotimchenkoBo, ZhangMaximilian, SeidlBo, PangMaximino, RedondoBogdan A., ŠolajaMayyada M. H., El-SayedBoguslaw, TomanekMazahar, MoinBojan, ŽunarMd Badrul, AlamBoMi, RyuMedha, RainaBonghee, LeeMeenakshisundaram, BalasubramaniamBonglee, KimMeghna, MehtaBoris, KurganovMei-Hsiang, LinBoris, PejinMeihua, YuBoris, DzantievMei-Ling, LiBotond, PenkeMelanie, SimpsonBozena, DenisowMelba C., JaramilloBradley, GrovemanMelgardt, De VilliersBradley, FergusonMelina, KerouBrandon, McKinneyMelissa, BirolBranka, Salopek-SondiMeng-Tsan, ChiangBrenda, WinkelMercedes, CuetoBrett, PhinneyMetin, UzBrian, WigdahlMette, ChristoffersenBrígida Ribeiro, PinhoMiaw-Ling, ChenBruce, BowermanMichael, HustBruno, BottaMichael, GreenBuddhadev, LayekMichael, TorzewskiByeong Hwa, JeonMichael, DanilenkoByung-Hoon, JeongMichael, NaumannC. James, McKnightMichael, TiemeyerC. Wyatt Shields IV, Shields IVMichael, HambloinCameron, PostnikoffMichael, MeirCamille, CarrollMichael, WoeltjeCândida Teixeira, TomazMichael, SchuppCankui, ZhangMichael, SchweizerCarla, FerreriMichael A., KennedyCarla, VarelaMichael F., HynesCarla, Assaf-BalutMichael K. E., SchaeferCarla, PereiraMichael W., LipscombCarla, BogaMichaela, SedlářováCarlos, CondeMichaela, WenzelCarlos, CaroMichaela, GriesserCarlos, MenesesMichał, BijakCarlos E., VelascoMichał, PikułaCarmela, ConteMichał, SwiecaCarmen, DinizMichel, KranendonkCarol, WadhamMichela, PieriniCarolina, OteroMichela M., GiulianoCassia S, MizunoMichele, GalluccioCastro-Rodríguez, DianaMichele, TonelliCatalin, VitelaruMichele, PritchardCătălin, AraniciuMichiel, Van Der VaartCaterina, MorciaMieszko, WieckiewiczCaterina, LeddaMiguel, HuesoCatherine, BelliniMiguel, CastanhoCatherine, ClellandMiguel, MartinezCátia, TeixeiraMiguel, TeixeiraCecilia, BrunettiMiguel Ángel, SolerCecilia, PozziMiguel Luiz Jr, BatistaCeferino A., CarreraMihaela Ileana, IonescuCelestine N., ChiMihai, NitulescuCélia F., RodriguesMihai V., PutzCelian, RomanMihir Kumar, MandalCelso, AlvesMikhail Y., InyushinCesar G., FragaMikhail M., M. Vorob'evCesario, Bianchi FilhoMilan, VranesChai-Lin, KaoMilan, UrbanChak-Sing, LauMilena, KordalewskaChandrashekhar, HonraoMilena, PetriccioneChang, DouMin, GaoChang Hoon, LeeMingdong, HuangChanghyun, RohMing-Fu, WangChangWoo, LeeMinghua, LiuChanning Judith, PallerMingming, TongChao, ChenMing-Wei, LuChaomin, SunMinjung, SongChaoyang, XueMinkui, LuoCharalambos, MichaeloudesMinli, XingCharles, HarringtonMirari, ArancibiaCharles W., SchindlerMireia, Martín-SatuéChe-Chen, ChangMiroslav, OveckaChe-Hsin, LeeMiroslawa, CichorekChe-Kun James, ShenMirza, BojićChen, FanMitali, TambeCheng, JiMitchell, LevesqueChenglin, MoMitsuru, ShinoharaCheng-Wei Tom, ChangMladen Roko, RašinCheng-Yang, ChouModar, KassanCheol, MoonModesto, Redrejo RodriguezCheryl L., PattenMohadetheh, MoulanaChi, ZhangMohamed Lotfy, AshourChia-che, ChangMohammad, SalehinChia-Jung, LiMohammad, NajlahChiaki, NishimuraMohammad Mijanur, RahmanChiaki, YoshikawaMohammed, Rafiq H SiddiquiChia-Li, YuMohan, GuptaChiara, VillaMohsin, KhanChiara, FalcianiMonica, GomaraschiChiara, MondelloMonica, GalloChiara, NadaiMonica, FedeleChi-Chung, WangMonica, FlorescuChiharu, SogawaMonika, Słomińska-WojewódzkaChih-Chien, ChuMonika, GarbowskaChih-Hsin, TangMonika, FuxreiterChih-Li, LinMonish Ram, MakenaChih-Wei, ChangMootaz, SalmanChih-Yung, TangMorgan, BeebyChi-Jan, LinMorgane, MichaudChikako, HondaMorkos A., HenenChi-Ming, WongMorten, NielsenChin-Soon, PhanMostafa, RatebChi-Tai, YehMotohiro, OkadaChiung-Cheng, HuangMotoyoshi, NomizuChlodwig, FranzMousumi, MajumderChongzhi, ZangMrinmoyee, MajumderChris, CavalarisMuhammad, ZafarChris, MaragosMuhammad, HanifChristiaan, Van OoijMuhammad, ZeeshanChristian, StockMuhammad Ayaz, AnwarChristian Peter, KubicekMuhammad Muzzammil, EdhiChristian R., Encina-ZeladaMukundan, RagavanChristine, EngelandMurali, DhanasekaranChristoph, BauerMurali, YandaChristoph, RaderMurray B., IsmanChristophe, HanoMuruganandan, ShanmugamChristopher, HadadMurugesan, ChandrasekaranChristopher H., DietrichMyron R., SzewczukChristopher M., SandersonMyung-Bok, WieChristopher N., LaRockNabanita, SahaChristopher P., MattisonNadège, JaminChristos K., KontosNadezhda, UstyuzhaninaChryssostomos, ChatgilialogluNadezhda A., GermanChuang-Rung, ChangNadezhda N., SushchikChuanlong, CuiNadine, NagyChun, WangNae, DunChunfu, XuNaïg, GueguenChung, WongNaofumi, ShimokawaChung-Hsin, WuNaohito, AbeChung-Yi, WuNaoki, MoritaChunhua, SongNaoki, TanakaChunyu, LiuNarasimham, ParinandiCindy, HodakoskiNatalia, ZhukovaCinzia, MontemurroNatália, MartinsCinzia Anna, VenturaNatalia V., ShcherbikClaire, JosseNatalie, HudsonClara, IannuzziNatalie, BorgClare, HoskinsNatalie P., HolmesClaude, Caron De FromentelNatalija, VelićClaude, LoverdoNathalia, Meireles Da CostaClaudia, RiccardiNathalie, VigneronClaudia, MaierNathaly, Reyes GarcésClaudia, Rangel-BarajasNathan, AlderClaudia, BustamanteNathan, BryanClaudia, Gonzalez ViejoNathaniel J., SzewczykClaudia, Luevano-ContrerasNaufal, ZagidullinClaudia, AlessandriNeha, ParayathClaudio, LuchinatNehru Viji, SankaranarayananClaudio, TaloraNelson, GomesClaudio, SlamovitsNenad, IgnjatovicCláudio, MaiaNéstor, Gutiérrez-MéndezCleber, AnconiNeval, YilmazClemens, Von SchackyNguyet-Thanh, Ha-DuongClemens K., WeissNicholas B., LarsonCleslei Fernando, ZanelliNicola, GiulianiClinton C., MacDonaldNicola, FestucciaClive, EvansNicola, MaureaClivel, CharltonNicola F., FletcherConcetta, Di NataleNicola M., CosentinoConstance, BaileyNicolai, KroghConstantin, CaruntuNicolas, Villegas-SepulvedaCornelia, BraicuNicolas, DestainvilleCornelis, SierNicolas, GouaultCorneliu, TanaseNicoletta, GaglianoCosima Damiana, CalvanoNicolò, MerendinoCostas, KiparissidesNidal, JaradatCraig L., FrankNikolai B., GusevCristiana, RadulescuNikolaos, GeorgopoulosCristiana, TanaseNikolaos, BouropoulosCristiane Damas, GilNikolaos, TsafantakisCristina, SisuNikolaos, LabrouCristina, AlcacerNikos, KodoudakisCristina, AguiarNikunj, SataniCristina, ClementNina, KamennayaCristina, MaccalliniNing, WangCristina, VenturiniNingning, ZhaoCristóbal, MezquitaNiraj, NiralaCsilla, BenedekNirmala, MavilaCyril, BourgeoisNishit, GoradiaD. Woodrow, BensonNobuhiro, NakamuraDaehwan, KimNobuyuki, TakahashiDainis, Rungis RuņģisNoelia, DuarteDaisuke, ObinataNorbert, De KimpeDamian, BartuziNorbert, GhyselinckDan, CojocNorio, SuzukiDana, MunteanNorio, YamamotoDana, HockovaNoriyuki, MuraiDana Maria, CopoloviciNoura, DosokyDaniel, MarcNuno, ValeDaniel, WeberNunzia, D'OnofrioDaniel, Piñero-DalmauNuria, Fernandez-GonzalezDaniel, Carvalho PimentaOh Seok, KwonDaniel, OhOi Wah, LiewDaniel, S. OhOldřich, FarsaDaniel, KracherOleg I., BaryginDaniel, GeisslerOleksandr, PokholenkoDaniel Alberto, SánchezOlga, Sánchez-PernauteDaniel A., TraylorOlga, GurskyDaniel A., KleierOlga, ViegasDaniel E., Martínez-TongOlga, KrizanovaDaniel Giddings, VassãoOlga, ZubkovaDaniel R., RoeOlga, RechkoblitDaniela, AlfanoOlga, KrestininaDaniela, De VitaOlga A., KoksharovaDaniela, MontesarchioOliver D., KingDaniela, GiacomazzaOlivier, PotteratDaniela, CaccamoOlivier, SchildgenDaniela, CarlisiOlivier, SorgDaniela, MarascoOlivier, CouxDaniela C., VazOliviero, CarugoDaniela Eliza, MarinOluwatosin, OluwadareDaniela Valeria, MinieroOmaima, NasirDaniele, VergaraOmer, YehezkeliDanielle, Majoor-KrakauerOnur, CilDaniil, OlennikovOronzo, BrunettiDarci, TraderOscar, Martínez-AlvarezDariusz, KarczOscar, Perez-MendezDariusz, ChlubekOscar, NunezDariusz Maciej, PisklakÓscar, Lorenzo GonzálezDavid, LibichÓscar A., Rojas RejónDavid, SchlaepferOury, CécileDavid, de Gonzalo-CalvoOxana V., GalzitskayaDavid, DrutovicOyuna, KozhevnikovaDavid, NicholsOzgul, GokDavid, HarrisP., Zavala-RiveraDavid, FrumanPablo, MurielDavid, FinkelsteinPablo García, De FrutosDavid, CoquerelPalanisamy, NallasamyDavid, KorasickPalanivel, GanesanDavid, MerklerPalmiro, PoltronieriDavid, BrookPaloma, Sánchez TorresDavid, BeveridgePalsamy, PeriyasamyDavid L., CheungPan, TaoDavid A., GellPaola, FossaDavid G., JacksonPaola, ZuluagaDavid R., WallacePaola, PiomboniDavid Ross, SibsonPaola, MaroniDavide, TosiPaola, FerrariDawn E, E QuellePaolo, GabrieliDebashish, SahuPaolo, TortoraDeborah, FratantonioPaolo, SeverinoDeepak, ParasharPaolo Armando, GagliardiDeguo, DuParagi, GáborDejan, AgićParis D. N., SvoronosDelgado, JosuePartha K, ChandraDelphine, FradinPascale, CharestDenis, GubinPaschalina, ChatzopoulouDenis I., BurchakovPashtoon Murtaza, KasiDenis S., KudryavtsevPasquale, RussoDerek, TomsPasqualina, D'UrsiDerek, BrazilPatrice, CoteDessislava, TodorovaPatrice A., MarchandDhaka Ram, BhandariPatricia, Fernandez-RobredoDhruba, SarkarPatricia, Gerbeau-PissotDi, LangPatricia, CarloniDiana, GasparPatricia, Guevara-FeferDiana, Hernandez-RomeroPatricia, BaldrichDiana E, RoopchandPatrícia, RijoDiane, ParkPatrícia, FaíscaDidier, MaryPatrick, BradshawDiego, HaroPatrick S., CalleryDiego, Muñoz-TorreroPatrizia, LoprestiDiego, CotellaPatrizia, RomanoDiego Franco, JaimePaul, KlineDiego Martin, BustosPaul, DalhaimerDiego Romano, PerinelliPaul, MischelDieter P., ReinhardtPaul, RaceDik-Lung, MaPaul C., TaylorDileep, VarmaPaul J, BrightonDimiter, AvtanskiPaul J., HigginsDimitri, GilisPaul Li-Hao, HuangDimitrios, LampakisPaul T., ThevenotDimitris, KardassisPaula, MoralesDipayan, SarkarPaula, Martins-LopesDivya, KamathPaulina, KosikowskaDivya, BhagirathPauline, SchaapDjuro, KosanovicPaulino, Gómez-PuertasDmitris, XirodimasPaulo, CarvalhoDmitry, VorontsovPaulo, MunekataDmitry B, ZorovPaulo José Gomes, CoutinhoDmitry B., ZorovPaulo Roberto, H. MorenoDoina, PiciuPavel, SemenyukDomenico, MontesanoPavel, KopelDomenico, Azarnia TehranPavla, BojarováDomenico, GarozzoPavleta, TzvetkovaDomenico, De BerardisPavlina, DolashkaDomenico, MarsonPavol, FarkašDonald, GullbergPaweł, BogdańskiDonald, SikazwePaweł, KonieczkaDonald, CameronPaweł, SobczakDonald H., BurkePayel, SilDonald T., YappPedro, FernandesDonata, FigajPedro Alexander, InfanteDonatien Pascal, KamdemPedro José, Martínez De PazDong, HanPeeyush N., GoelDong, XuPei-Ming, ChuDongchul, KangPeishen (Elva), ZhaoDong-Hun, BaePei-Shiue, TsaiDorina, LauritanoPejin, BorisDorota, ŻelaszczykPemra, DorukerDouglas, KojetinPeng, XuDragana, NikitovicPengcheng, WangDragos, HorvathPenny, BeuningDrake, MitchellPeppino, MirabelliDubravka, Vitali CepoPer, MalmbergDuhyung, ChoiPer, HedegårdĐurđica, AčkarPereira, RubenDzmitry, ShcharbinPeta, HarveyEdgar, MarquezPeter, StathopulosEdit, CsapoPeter, HasenhuetlEdoardo, ParrellaPeter, PolčicEdouard, KraffePeter, RichardEduard, JirkovskyPeter, WilsonEduardo, Lopez-ViñasPeter, LipkeEduardo, GuzmánPeter, GronesEduardo, AnsorenaPeter, McCafferyEduardo A., EspesoPeter, GoettigEduardo De Jesus, OliveiraPeter, OlingaEdvin, PozharskiyPeter, ShepherdEdward, O’BrienPéter, LöwEdward, FilardoPéter, GyulaEdward H., SharmanPeter J., RoyEdyta, WieczorekPeter T., CorbettEdyta, ReszkaPetr, ChlandaEggehard, HollerPetr, HrdlickaEgor Y., PlotnikovPetr, KarlovskyEhab, Abdel-LatifPetr, ZamostnyEiji, YubaPetra, ProchazkovaElah, PickPetra, HaenzelmannElena, CicheroPetranel, FerraoElena, CatanzaroPetros, GiastasElena G., BioscaPhil, WhitfieldEleonora, CominelliPhilip, KitchenElfriede, BacchiPhilip, CarellaElia, RanzatoPhilip, CalderEliane, De Oliviera SilvaPhilipp, HohensinnerElinor, HortlePhilippe, DeschampsElisa, BustaffaPhillip M., RendleElisa, CairrãoPhillip T., HawkinsElisa, KorenblumPhiwayinkosi V., DludlaElisa, FrezzaPhurpa, WangchukElisabetta, CaiazzoPickrell, AliciaElizabeth, VafiadakiPierantonio, De LucaElizabeth, SkellamPiero, SestiliElke, MarkertPierre, VacherElsa, Gongora CastilloPierre, GenevauxElton, ZeqirajPierre-Antoine, BonnetElwira, ChrobakPietro, ScicchitanoElżbieta, BudziszPietro, Di FazioEmanuel, RaschiPilar, López-CornejoEmanuel Karl, PeterPilar, Serra-AñóEmanuela, BostjancicPin-Der, DuhEmanuela Prado, FerrazPing-Jyun, SungEmanuele, MontomoliPio, ContiEmil, AlexovPiotr, ŚwiątekEmilio, HirschPiotr, RychahouEmma, LangellaPiotr, KoprowskiEmma, PerssonPooyan, MakvandiEmma, AnderssonPradeep, TyagiEmmanuel, PanterisPramod, YadavEmmanuel, BourdonPranabananda, DuttaEnrica, De RosaPranay, SrivastavaEnrique, Domínguez ÁlvarezPrashanth N, SuravajhalaEric, RuellandPrashiela, MangaEric, SimanekPratip, BhattacharyaEric, DietelPraveen L., PatidarEric B., KmiecPreethi, UdagamaEric S., GoetzmanPriya R., BanerjeeErica, SilvestrisPriyanka, KothariErik, KarlssonPrzemyslaw, PlonkaErik T., YuklPubudu, HandakumburaErina, VlashiPugazhendhi, ArivalaganErlinda, TheQi, TanErwin, BohnQing, JiangEstela L., ArreseQingbao, DingEsther, MahabirQingbin, CuiEsther, BarreiroQingxiang, ZhouEsther, TamayoQinZi, XuEstrada-Bernal, AdrianaQiu-Xing, JiangEstrella, SantamariaQuentin, SchmetzEtsu, TashiroQX, MuEugenia, BezirtzoglouR. Jurgen, DohmenEung-Kwon, PaeRachel, HeveyEusebio, ChiefariRachel, WellsEva, KnochRachid, LahlilEva, KatonaRachita, YadavEva, SahakianRadhakrishnan, PanatalaEva E., Rufino-PalomaresRadivoje, ProdanovicEva M., GalvezRadosław, BonikowskiEvanna, MillsRadoslaw Kajetan, KowalskiEvgeni Yu., ZerniiRafael, Guillén-BejaranoEvgeny, ApartsinRafael, PeñafielEwa, MarcinkowskaRafael C., BernardiEwa, Ostrowska-LigęzaRafal, BartoszewskiEwa, Wałecka-ZacharskaRaffaele, FrazziEwa Anna, BienkiewiczRaffaele, D'AmelioEwelina, KrólRaffaele, CapassoEzequiel, TolosaRaffaele, RiccòEzio G., GiacobiniRaffaella, MicilloFabio, FaragunaRahman, Rahman PourFabio, BoylanRahul, JayantFabio, GanazzoliRahul, TyagiFabio, GentiliniRaimundo, FreireFabio, PennaRainer, NikolayFabio, VeronesiRainer W., BussmannFabrizio, GentileRajan, LamichhaneFabrizio, CalderaRajendram, RajnarayananFahim, AhmadRajesh, KomatiFara, Brasó-MaristanyRajesh K, HarijanFarid, RahimiRajib, MukherjeeFatiha, NassirRakesh, UpadhyayFatima, RivasRakesh K., TiwariFatima, CerqueiraRakesh K., UpadhyayFawzia, Bardag-GorceRalf, GaebelFederica, PollastroRalph, RuehlFederica, PasiniRamesh, AdhikariFederico, FogolariRamon, BatistaFederico Alessandro, RuffinattiRan, DroriFei, MoRaphael, StollFei, HeRaphael, CanadasFelicia, GohRaphael, SchneiderFelix, HauschRaquibul, HasanFeng, RaoRashmi, ChandraFengjie, SunRaul, Torres-RuizFenglong, WangRaúl, VinetFenny, DaneRaúl, Ferrer-GallegoFerdinand, MarletazRavikanth, NanduriFerdinando, NicolettiRavinder, NagpalFerdinando, BonfiglioRavis, ZalubovskisFerdinando, MannelloRay-Bing, ChenFerenc, ErdődiRazieh, RafieeniaFernandez De, Simon BrigidaRebeca, BustoFernando, LealRebeca D., Martínez-ContrerasFernando, AlbericioRebecca, CreamerFilomena, FonsecaReddy M, PabbidiFlaviana, Di LorenzoReena, BapputtyFlavio, RizzolioRegina, Fölster-HolstFlorencia, Di PietroRemington, PoulinFlorent, FigonRemy, PoupotFlorian, AltegoerRenata, MikstackaFlorin George, HorhatRenata, KontekFozia, SaleemRenata, CiccarelliFranca, RossiRenate, BurgerFrancesca, VasileRenato, HeidorFrancesca, GattoRené, CsukFrancesca, GoriniRenjie, TangFrancesco, FedeleRenren, BaiFrancesco, AnielloRian, GriffithsFrancesco, MilanoRicardo, BoschFrancesco, TrottaRicardo, DiasFrancesco A., AprileRiccardo, SarzaniFrancesco M., RestivoRiccardo, FerroFrancesco Paolo, BusardòRichard, ZuelligFrancine, Perrine-WalkerRichard, HrabalFrancisca, BlancoRichard, DaniellouFrancisco, LesRichard E., DavisFrancisco, LeonRichard M., AnthonyFrancisco, Cen-PachecoRichard P, NovickFrancisco, MeijideRieko, NakataFrancisco, Cabrera-ChávezRimantas, DaugelaviciusFranciska, ErdőRita, MorigiFranck, MartinRita, López-CebralFranco, GozzoRita, TeixeiraFrançois, NoelRitin, SharmaFrançois, MarceauRitva, TikkanenFrançoise, Lohézic-Le DévéhatRobersy, SánchezFrançoise, BafortRobert, BjorklundFrank, AlexisRobert, SafirsteinFrank, TackeRobert, FlavellFranka, Klatte-SchulzRobert, JerniganFranko, BURCULRobert, WojciechFranny, JongbloedRobert, SotoFrantišek, BaluškaRobert, RuckerFranz, WorekRobert, SchneiderFranz, RoedelRobert L., FurlerFranziska, Roth-WalterRobert J., BrownFritz, BoegeRoberta, GaleazziFuchao, XuRoberto, LuisettoFuguo, LiuRoberto, Fernandez-LafuenteFumio, MatsudaRobyn Laura, KosinskyFumiyoshi, MyougaRoch-Philippe, CharlesG. Ekin, Atilla-GokcumenRocio Alejandra, Chavez-SantoscoyG. Jason, SmithRodica-Mariana, IonGábor, RigóRodrigo, LacruzGabriel, MarcRoger A., MooreGabriela, Castañeda-CorralRogerio, Sotelo MundoGabriele, CandianiRogerio, RibeiroGabriele, CarulloRohan, SamarakoonGabriele, ToiettaRohitash, JamwalGabriele, MichelettiRoland, VollgrafGabriele, BajRolf, TeschkeGabriella, D’OraziRoman, V. KholodenkoGabriella, BakiRoman, PaduchGadi, PelledRoman, JakobGaetano, SantulliRoman B., LesykGandhi, Rádis-BaptistaRonald, HillsGang, LiuRonald B., BrownGareth, TribelloRong-San, JiangGary, LoughranRosa, Lemmens-GruberGaurav, GhagRosa Maria, PerestreloGaurav, RaikhyRosália, SáGaurav, MehtaRosario, DonatoGautam, SethiRossana C., ZepedaGaynor E., SpencerRothwelle J., TateGeetesh K., MishraRozenn, Le HirGema, Mendez-LagaresRuchi, ShahGennaro, EspositoRuchira, ChatterjeeGennaro, RiccioRudiger, WoscholskiGenta, KakiyamaRudimar Luiz, FrozzaGeoffrey, MuellerRui M. Gil, Da CostaGeoffrey I, McFaddenRui Manuel Santos Costa, De MoraisGeorge, LambrinidisRun, ZhangGeorge, AnifandisRuo-Kai, LinGeorge, HeathRupesh, DeshmukhGeorge, KomisRuth C., BorghaeiGeorge Cătălin, MarinescuRyan, GartenGeorge-Albert, KarikasRyan A., HenryGeorges, DreyfusRyszard, AmarowiczGeorgios, TsaniklidisRyuichi, SakaiGerald, SoslauRyutaro, AsanoGerd, PrehnaSabina, GórskaGerhard, BrausSabina, Kędzierska-MieszkowskaGerhard, ErkelSabrina, PriclGerrick, LindbergSabrina, Di BartolomeoGerrit, BegemannSabrina, BimonteGerry, HammondSachiko, Toma-FukaiGertrud, LundSachin, TeotiaGerwin, HellerSachin, KotakGhazala, MustafafSadia, SaifGheorghe, IliaSaeid, HosseinzadehGiacomo, SaielliSagrario, Martín-AragónGiampiero, CaiSaheem, ZaidiGiancarlo, GrossiSaianand, GopalanGiandomenico, CorradoSailen, BarikGianfranco, GennariniSaivageethi, NuthikattuGianluca, BossiSalam, IbrahimGianluca, PicarielloSalik, NazkiGianluca, MaffeiSalvatore, TerrazzinoGiau, VoSalvatore, PepeGill, DiamondSamantha, MessinaGiorgia, MoriSameh S. M., SolimanGiorgio, GallinellaSamuel, GanGiorgio, LenazSamuel, Garcia PerezGiosuè, AnnibaliniSamuel J., WebbGiovanna, RomanoSamuel K., CamposGiovanni, MonacoSancho-Bru, PauGiovanni, BubiciSandeep K., MishraGiovanni, GrignaniSandipan Roy, Roy ChowdhuryGiovanni, ValliniSándor, GóbiGiovanni, GalassoSandra, LindstedtGiovanni, D'OrazioSandra, LeiszGiovanni, La Penna Sandra, TorrianiGirish, PattappaSandra, Martínez-TuriñoGirolamo, PelaiaSandra, PucciarelliGiulia, BianchiSandra, Garcia-GallegoGiulia, GavaSandra B., GabelliGiuliana, MuzioSandrine, Cammas-MarionGiuliana, MagnaccaSandrine, BouquillonGiuliano, SiligardiSandro, Fernandes Da SilvaGiuliano, BernalSandro, ArguellesGiulio, VernaSang Bing, OngGiulio, CavalliSangchun, ChoiGiuseppe, ScherilSangho, LeeGiuseppe, LazzaraSanjay, GuptaGiuseppe, AlloattiSanjay, VarikutiGiuseppe, ZagottoSan-Lang, WangGiuseppina, CatanzaroSantiago, Mora GarciaGiuseppina, AdilettaSantiago, Martínez-CalvilloGladius, LewisSantiago, Ruiz-MoyanoGlaucia, Paranhos-BaccalàSantosh, DhakalGloria, BonuccelliSantosh, GhoshGloria María, Molina SalinasSara, ParrettiniGokhlesh, KumarSarah, ChappleGolam Mezbah, UddinSarah D., LinnstaedtGong-Hong, WeiŠárka, PetrováGordana, Wozniak-KnoppŠárka, KubinováGordana Nedic, ErjavecSathish Kumar, NatarajanGow-Chin, YenSathyanarayanan, Sevilimedu VeeravalliGracia, Merino PelaezSatish Kumar, NoonepalleGraham, McCloreySatoshi, NakataGrażyna, JanikowskaSatoshi P., TsunodaGreg, WangSatu, KuureGreg M., ThurberSaurav, SarmaGregory, WiedmanSawsan A., ZaitoneGreta, BalciunieneScott D., PeganGrigory, MaksaevSean, EganGrzegorz, GrynkiewiczSean, RuddGrzegorz, ZagułaSebastiaan, MeijsingGuadalupe, Gómez-BaenaSebastian, PohlGuang, HuSebastian, PonsGuangdong, YangSebastian L., RiedelGuan-Jhong, HuangSebastiano, IntagliataGuannan, ZhangSébastien, BesteiroGuannan, HuangSeema, SaksenaGudrun, StenbeckSei, KakinumaGuennadi, SaikoSek-Man, WongGuglielmo, VeronaSen, TakedaGuido, KeijzersSeonyeong, KwakGuido, GessnerSeraina, FaesGui-Rong, ZhangSerdar, DursunGunhild, Von AmsbergSerge, NatafGunnar, JeschkeSergej, OstojicGunnar, DittmarSergey, VyazovkinGunter, SchmidtkeSergey, KozlovGuo Jun, LiuSergey A., AkimovGuolin, ZhangSergey M., EfremovGustavo, MonneratSergio, MartínezGustavo Alberto, ChiabrandoSergio, Granados-PrincipalGvozden, RosicSergio, Cuevas-CovarrubiasGyuhwa, ChungSérgio F., SousaHafiz Ansar Rasul, SuleriaSergio Ruffo, RobertoHafiz M. N., IqbalSeung Pyo, GongHai-Chou, ChangSeunghwan, YangHaili, ChengSeung-mann, PaekHailong, SongSeyun, KimHaitao, ShiShafiul, AlamHai-yang, LiuShailendra, MauryaHaizhen, ZhongShan, Ali ZaidiHak Joong, KimShang-Te Danny, HsuHamidullah, KhanShani, Shenhar-TsarfatyHan, XiaoShanon L., CaspersonHan-A, ParkShaoyong, LuHang, TaShashank, KulkarniHang Fai (Henry), KwokShau-Wei, TsaiHan-Gang, YuShazia, JamshedHanna, Bis-WencelSheldon, RowanHans-Arno J., MüllerSheng-Nan, WuHao, SunSheng-Shih, WangHao, HuangShereen, SabetHarald, LoppnowSherif T.S., HassanHarini, MohanramShiao-Wen, TsaiHarm J., HeusinkveldShida, YousefiHarsha, KariyawasamShigeki, HabaueHartmut, KuhnShigeru, SatohHaruki, HoikeShihori, TanabeHassan M. A., HassanShikha, SainiHavovi, ChichgerShin, TakasawaHayssam M., AliShin-Hyung, ParkHeather, MortiboysShin-ichi, KayanoHee Jun, ChoShinichiro, OchiHee Y., KangShinji, YokoyamaHeeyoung, ChaeShinji, OhtaHehuang, XieShinya, SuzukiHeikki, VuorelaShiping, TianHeiko, LangeShiqiang, GaoHelder, MarcalShirin, BonniHelder, MaiatoShona, MurphyHelena, Kupcova SkalnikovaShosuke, ItoHelena, Prima-GarcíaShuai, GaoHelene, DumondShuang, LiuHelgo, SchmidtShuang-Qing, ZhangHema Prasad, NarraShuhua, XiHemant, GiriShujia, ZhuHenning, PoppShulan, FuHenry, ShumShu-Lan, YehHenry I., Castro-VargasShun-Fa, YangHerbert, VenthurShusen, ZhengHerman H. W., SilljéShu-Yao, TsaiHerwig, MollSiemowit, MuszyńskiHetron Mweemba, Munang’anduSifan, ChenHidekazu, HiroakiSilvano, FerriniHideki, KawashimaSilvia, MarchesanHideki, KawasakiSilvia, DAnteHideki, SakaiSilvia, Di AgostinoHidetoshi, TaharaSilvia, ProiettiHidetoshi, InagakiSilvia Vincenzetti, VincenzettiHideyuki J., MajimaSilvio, PanettieriHikaru, SuenagaSilvio, Vaz JuniorHilal, ZaidSimon, HigginsHimanshu, KathuriaSimon, EbbinghausHiroaki, SaikaSimona, PaceHiroaki, KitagishiSimona, LattanziHirofumi, EnomotoSimona, RapposelliHirofumi, ShimomuraSimona, CollinaHirofumi, InoueSimona, Delle MonacheHirofumi, SawaiSimone, Di MiccoHirofumi, KaiSimone, MoserHironori, KatoSinead, WeldonHironori, YoshinoSiumara, AlcântaraHiroshi, TakemoriSiyang, LengHiroshi, WadaSkander, ElleucheHiroshi, IsobeSmaoui, SlimHiroyuki, KitahataSmaranda Dafina, OnigaHisakazu, OgitaSocorro, Retana-MarquezHisashi, TakeuchiSofia, SousaHisashi, IzasaSofia, AvnetHomaira, RahimiSofia, AgriopoulouHong, MaSokol, TodiHongbing, LiuSolène M., EvrardHonghui, GuoSonal, SinghHongliang, HeSonali, BhattacharjeeHong-Seok, SonSong, Beng KahHooi Hooi, NgSong-Tao, LiuHoracio, Pérez SánchezSonia, MediouniHo-Shik, KimSonia, ScarfìHosseinali, AsgharianSonia, MedinaHou-Jin, LiSonja, SucicHouliang, TangSophie, BlatHouyang, ChenSophie, AyciriexHristo, PenchevSoren, ToxvaerdHsin-Chieh, LanSotiris, AmillisHsiuying, WangSoumyaroop, BhattacharyaHu, ChenSpiros S., SkourtisHua, GongSravan Kumar, PatelHuanzhong, WangSrinivas, NandanaHubert, AntolakSrinivas, MutalikHugh Douglas, GooldSrinivas, SomarowthuHugo, RochaSrinivas, AbbinaHui-Chi, HuangStanislav, RybtsovHuiquan, LiStanisław, WołowiecHung-Chung, HuangStankevičius, VaidotasHuzefa, RajaStavros, LalasHyun, Ah KimStefania, CaparrottaHyun-Woo, RheeStefania, PollastroIain, HargreavesStefanie, ScheuIan, CollinsonStefano, PieracciniIan, MartinsStefanos, MourdikoudisIan, LianStefka D., SpassievaIan M., JonesStephan, BlockIan C., SchneiderStephan Joel, ReshkinIan M., SimsStéphane, TéletchéaIan Max, MøllerStephanie, SisleyIbarra-Alvarado, CésarStephanie, GantzIbrahim M., Abu-ReidahStephen, AdelsteinIchiro, MaruyamaStephen, LeonardIdolo, TedescoStephen A., RenshawIgnacio, FernándezStephen C., AllenIgnacio, Mir SanchisSteve, BourgaultIgnacio, Moya-RamírezSteve, LeuIgor, PottosinSteve, WhittenIgor, ZhukovSteven M., PascalIgor, CesarinoSteven R., Van DorenIhosvany, CampsSubhadeep, ChakrabartiI-Ju, YehSubramanya, SreeramaIkechukwu P., EjidikeSu-Hyung, HongIkram, BelghitSujit, BasakIlaria, RussoSumit, AgarwalIlaria, LaurenzanaSung-Hoon, KimIleana C., FarcasanuSung-Kun, KimIlka, AxmannSuowen, XuIl-Kwon, ParkSuprit, GuptaIlse, DaehnSuresh, PannerselvamImaël Henri Nestor, BassoléSurtaj, IramIn Soo, YoonSusana, CampuzanoIndika, EdirisingheSusana, CardosoIndira, ShrivastavaSusana M., CardosoInes, MármolSusana N., SilvaInes, DelfinoSusana Santos, BragaInmaculada, SeguraSusanne, SattlerIo, AntonopoulouSusanne, BaldermannIoana, DemetrescuSushanta, GhoshalIoanna-Katerina, AggeliSvenja, SydorIoannis, LignosSvetlana, DeryushevaIoav, CabantchikSvetlana, TamkovichIolanda Midea, CuccoviaSvetlana P., ChapovalIrena, RotermanSyed Haris, OmarIrene Russo, Russo KraussSylva, PrerostovaIreneusz, OchmianSylvain, FisetIrina, GushinaSylvain, JeandrozIrineu, BatistaSylvie, HermouetIrwin Rose Alencar, MenezesSylvie, BabajkoIryna, LebedyevaSylvie, RebuffatIsaac, OnyangoSylvie, FriantIsabel, AndiaSyn, YeoIsabel, Iriepa CanaldaSzczepan, BednarzIsabel, HaroSze-Ming Mildred, YangIsabella, PanfoliSzilvia, BőszeIsao, IshiiSzymon, KaczanowskiIsidoro, FelicielloT. Doohun, KimIsrael S., IbarraTad A., HolakIto, FumiakiTadeusz, InglotIulian, AntoniacTaeho, KwonIuliana, AproduTae-Hong, KangIva, BoušováTakaaki, InadaIvan, SalmerónTakahiro, KannoIván, MontenegroTakahisa, KanekiyoIván, Gómez OcampoTakako, YasudaIvana, MarekovićTakamitsu A, KatoIvana Generalić, MekinićTakashi, MatsuiIvana Vinkovic, VrcekTakashi, TodaIvo, GrabchevTakayoshi, WakagiIzabela, ZakrockaTakehito, NakazawaJ. Catharina, DuvigneauTakemi, OtsukiJ. Richard (Dick), McIntoshTakuji, TanakaJ.D., Flores-FélixTakuya, YoshidaJacek, AntonkiewiczTanaya, LahiriJacek, OtlewskiTânia, MeloJacek, WilczyńskiTanima, BoseJacky Y., SuenTanja, GrkovicJacqueline, VitaliTao, ZhangJadwiga, HandzlikTao, PanJae Sue, ChoiTao, WangJaegyun, OhTara, DavisJaehyeon, OhTara H.W., DobsonJae-Min, OhTatiana, El-BachaJaewhan, SongTatiana I., SamoylovaJaime, García-MenaTatjana, RadosavljevićJaime, EscalanteTatyana V., SavchenkoJaiprakash, SharmaTeet, SeeneJakob, FrankeTej Narayan, PoudelJames, LillekerTeng, PengJames, La ClairTeodora, BavaroJames, FackenthalTerry, GolombickJames, AdamsTessa J., BarrettJames B, AmesTetsumi, IrieJames B, BurrittTetsuro, TamakiJames C., GumbartTharin, BlumenscheinJames K., BashkinTheo, PlantingaJames P., NakasTheocharis C., StamatatosJames P., HardwickTheodoros, EleftheriadisJames R., FuchsTheresa, HautzJan, BenadaThiagarajan, VenkatesanJan, CabriThiago Luiz, Alves E SilvaJan, PaczesnyThissa N., SiriwardenaJan, SimoniThomas, BarthJan, KovárThomas, GreitherJan, JanouškovecThomas, MüllerJan, MazerskiThomas, PollardJan, KorabecnyThomas, ZülligJan, MalinskyThomas, BrettJan, ŁyczakowskiThomas, DeCourseyJán, KyselovicThomas, CaspariJan A., MolThomas, KloseJan Henrik, FinkeThomas W., BellJan M.C., GeunsThorsten, KraskaJana, FrankováTianfang, WangJana, OklešťkováTianjiao, JiJand Venes, MedeirosTiffany, CazaJane, AntonyTiit, LukkJane E., IshmaelTimo, SorsaJanet, NewmanTimofey, RozhdestvenskyJanmes, SpencerTimothy, EganJanusz, NowickiTimothy R., HooverJarmila, JedelskáTimur Yu., MagarlamovJaromír, MyslivečekTina Rise, TuvengJaroslav, PelisekTingting, HongJarosław, CzyżTitu, DevamaniJarosław J., SakTiziana, PandolfiniJasbir, UpadhyayaTobias, MadlJasenka, CosicTom L., BroderickJasna, JablanTomáš, HrnčířJason, BodilyTomasz, PniewskiJason M., BelitskyTomasz, CzechowskiJaume, TorresTomasz, PoplawskiJavad, Sharifi-RadTomasz, SliwinskiJavier, Martinez-UserosTomasz, WróbelJavier, Molina-CerrilloTomasz, NiedzielaJavier, Garcia-PardoTomasz, ZapolskiJavier, BrumosTomasz, BondaJavier, TerolTomasz, JaroszJavier, CerezoTomasz, PowrózekJavier A., Ceja-NavarroTomasz, HuraJay, FoxTomasz M., KarpinskiJayachandran, VenkatesanTomoharu, TakeuchiJayarama, GunajeTomohiko, TsugeJaylyn, WaddellTomohisa, TodaJean Christopher, ChamcheuTomonari, TanakaJean Luc, OlivierTomoya, ImaiJean Michel, MaixentTomoyuki, MukaiJean-Claude, AssafTorgils, FossenJean-marc, LancelinToshikazu, KawaguchiJeff, BronsteinToshiro, AraiJeff, SchwinefusToyonobu, UsukiJeffrey, FillinghamTracy Murray, Murray StewartJeffrey, BrenderTran Dang, XuanJency, ThomasTricia, Van LaarJender, WuTristano, PancaniJeng-Wei, LuTrude, WicklundJenifer C, CroceTrudi, ColletJennifer, VandoorenTsung-Hsien, LeeJennifer, HaynesTsuyoshi, ShimoJennifer, DeLucaTsvetko, ProkopovJennifer, HulingTugba, KucukkalJennifer, LachowiecTuli, MukhopadhyayJennifer R., McCallTzong-Shyuan, LeeJens, HarderTzu-Fan, WangJens, AppelTzu-Ping, KoJens O., LagerstedtU Gianfranco, SpizzirriJen-Tsung, ChenUlises, Páramo-GarcíaJeongmi, LeeUlrike, BaschantJeremie, TopinUmesh, BadrisingJeri-Anne, LyonsUroš, GašićJerod, DentonUrsula, StrandbergJeroen, PolletUrszula, Gawlik-DzikiJeronimo, BravoV. Prakash, ReddyJesper Thorvald, TroelsenVaibhav, JainJesse, GreenerVaibhav, MundraJessica, WalkerValentin, CracanJesus, Jiménez-BarberoValentina, PavicJesus, SimalValentina, PallottiniJesus, De La CruzValentina, SalaJesus, CirizaValentina, CecariniJesús, Cosín-RogerValério, Monteiro-NetoJi Hoon, JungValtcho, ZheljazkovJia, ZengVan Der Wal, PeterJiajia, SongVan G., WilsonJian, ShiVânia, MoreiraJianjun, WangVas, PonnambalamJiann-Ruey, HongVasanthy, NarayanaswamiJianyang, DuVasil, GeorgievJianyong, LiVasil, SimeonovJiao Jiao, LiVasileios, ZiogasJie, SunVasiliki, SiozopoulouJie (Jane), WangVasiliki, LagouriJihua, YuVasilios E, PapaioannouJimmy T., EfirdVasily, SvetashevJine, LiVassya, BankovaJiney, JoseVello, TouguJing, SuVenkateshkumar, PrabhakaranJing, TangVera, JencovaJingbo, XiaVeronica, San-MiguelJingjing, SunVeronica, AntipovaJingshu, GuoVeselin R., MaslakJinhee, ChoiVicente, RubioJinkai, YuanVicky, SophianopoulouJinku, BaoVictor, GoncalvesJinnan, ZhangVíctor, García-GonzálezJinxiang, WangVíctor, Quesada PérezJiongwei, WangVidhya, RaoJiranuwat, SapudomVidya, ChandrasekaranJitendra, Kumar SainiVidya, Mangala PrasadJiujiu, YuVidyasiri, VemulapalliJi-Yeong, BaeVijay, RamakrishnanJoachim, StorsbergVijay, KumarJoachim, GeyerViji, DraviamJoanna, WojtkiewiczVilma, PetrikaiteJoanna, Achinger-KaweckaVilma, KaškonienėJoanna, BojarskaVincent, HumblotJoanna, Kolniak-OstekVincent, BeringueJoanna, DepciuchVincenzo, LionettiJoanna Izabela, LachowiczVinicius Costa, GalvãoJoanne, JamieVioleta S., RilchevaJoão Antônio Leal, De MirandaVishal, GohilJoão Paulo, FabiVisvaldas, KairysJoaquim, VivesVita, GolubovskayaJoaquin, Manzo-MerinoVitalba, RuggieriJoaquin, RodrigoVitaly A., SorokinJoaquín, García-EstañVittorio, CapozziJocelyn, BescondVittorio, CapuanoJoel, Corrales–GarcíaVittorio, BianchiJoel C., WattsViviana, CostaJoel M., HarpVlad, MuresanJoelle, AmédéeVladimir, TrajkovicJoep C., DefescheVladimir, KubyshkinJogender, MehlaVladimir, ReukovJohanna, KlughammerVladimir, VladimirovJohannes, NotniVladimir, TitorenkoJohn, ManthyVladimír, ChobotJohn, WestbrookVladimir I., VladimirovJohn E., GatesyVladimir V., KuznetsovJohn J., MilledgeVladimir V., TolstikovJohn K., CraneVladimíra, KňazovickáJon, AshleyVolodymyr, RadchukJonas, FranssonVytas, ŠvedasJonathan, PhillipsW.S. Fred, WongJonathan, ClarkeWade, RussuJonathan, SchislerWakako, TakabeJonathan, BissonWalid, ElfallehJonathan, BackerWalter, WahliJonathan, McMurryWatson J., LeesJone A., StanleyWayne, CarterJong Won, HanWayne F., ReedJoo Hyoung, LeeWeaam, EbrahimJoonseok, OhWei, DuanJordi, CampsWei, ZhuJordi, ErasWei, ZouJörg, OpitzWei, LeiJorge, EstévezWeicai, ZhangJørn B, ChristensenWei-Chyou, WeiJose, Sanchez-SalasWeidong, WangJose, Rodríguez-MoratóWei-Dong, HeJose, TudelaWei-En, YuanJosé, QuintanaWeiheng, SuJosé, Caparros-MartinWeijie, ZhouJosé, SilvaWeiqin, ChenJosé, MancheñoWei-sheng, FengJosè, Divino-FilhoWeiwei, XueJose Antonio, Poveda LarrosaWei-Yu, ChenJosé Antonio, Morales-GonzálezWen, ShiJosé C.S., Dos SantosWenjing, WangJose Carlos, Jimenez-LopezWentian, WangJosé Dijair, AntoninoWen-Wan, ChaoJosé Guadalupe, Soñanez-OrganisWenwei, ZhengJosé Hipólito, Isaza MartínezWen-Wei, ChangJosé I., LópezWen-Wu, LiJose Joaquin, MerinoWiesława, RudnickaJosé L., NeiraWilfried Le, GoffJose M., Romero-EnriqueWilhelm, HuisingaJosé M., Álvarez-SuarezWioletta, SamolińskaJose Manuel, LozanoWojciech, KozdruJose Manuel, AgeitosWojciech, SmułekJosé Manuel, Rodríguez-PérezWolfgang, SchulzJosé Manuel Pérez, De La LastraWolfgang, DubielJosé María, AlundaWolfram, KunzJosé Pedraza, Pedraza ChaverriWoong June, ParkJosef, JiricnyWujin, SunJosep, Esteve-RomeroXiang Simon, WangJosep, CollXiangbing, MengJosep, MercaderXiangsheng, LiuJoseph, TaubeXianping, ChuJoseph, LevineXiaobo, WangJoseph, ZaiaXiaochen, HeJoseph, GovanXiaochun, YangJoseph, SottnikXiaogang, DuJoseph, LorentXiaoguang, LiJoseph Calvin, KouokamXiaohui, YanJoshua, WaxmanXiaojun, NieJosip A., BorovacXiaoling, LengJosipa, VlainićXiao-Ning, ZhangJuan, ZhouXiaosheng, WangJuan, Segura-AguilarXiaowan, WangJuan, ValcarselXiaoxia, LeJuan Antonio, NavarroXiaoyan, ChengJuan A., Castillo-GaritXiaoyan, ChenJuan C., NavarroXiaoyu, ZhangJuan Carlos, García-RamosXiaoyu, WengJuan J., Giner-CasaresXin, WenJuan Miguel, Soria GarciaXinbo, GuoJudit, RemenyikXingcheng, LinJudit, VáradiXinggui, ShenJudita, KinkorováXingguo, LiangJudyta, Cielecka-PiontekXingrong, DuJuei-tang, ChengXinsheng, JuJuhyung, KimXinxing, ZhangJui-Yang, LaiXu, CaoJui-Yen, HuangXu, BinJules, ThibaultXuechu, ZhenJulia, ShackelfordXuntian, JiangJúlia M.C.S., MagalhãesYan, LiJulia O., FedotovaYang, MeiJulien, Saint-PolYang, ZhangJulien, TailhadesYanlin, YuJuliette, Aury-LandasYan-Shen, ShanJulio, CaballeroYanxi, LiJulio, MoralesYao, TangJun, RenYasir, SuhailJun, SuzurikawaYasuhiko, MatsumotoJun, LuYasuhiro, OzekiJung-Hwan, KimYasukazu, DaigakuJunguk, HurYasuko, TanakaJunhui, JiYasumichi, InoueJunjie, FuYasuyuki, IshikawaJun-O, JinYaxia, YuanJunsei, TairaYee Ling, WuJunyan, TaoYehuda, ShienfeldJunzeng, ZhangYeon S., HanJurgen, EngelberthYeonseok, ChungJürgen, BrosiusYeranddy A, AlpizarJurica, ŽučkoY-h., TaguchiJustin, MobleyYi, ShenJustyna, CybulskaYi Tzu, ChoJustyna, Rosicka-KaczmarekYibin, FengJustyna, Stefanowicz-HajdukYibin, YangJutta, SchöpferYifat, MillerJyung-Hurng, LiuYigal, CohenKagan, KermanYi-Han, ChiuKai, WangYi-Hsien, HsiehKaido, KurrikoffYin, SunKamakoti, BhatYing, WangKamal, ChowdhuryYing, LuKamalendra, SinghYingnian, WuKamayani, SinghYingqian, ZhanKamil, KucaYinnian, FengKaminský, JakubYiqiang, ZhanKamyar, ZahediYixiang, ZhangKandasamy, SaravanakumarYixuan, ZhangKankan, WangYi-Yuan, YangKapil, MoothiYmera, PignochinoKapilkumar, PatelYoel, KashmanKaren C., GlassYogendra, MishraKari, StoneYogendra, KanthiKari L, StoneYoichi, ImaiKarnaker R., TupallyYoko, MatsudaKarolina, SłoczyńskaYoko, ItakuraKarolina, Szewczyk-GolecYoko, HirataKarthikraj, RajendiranYonatan, DubiKasturi, PalYongchan, KwonKasturi, RoyYonggong, ZhaiKatarzyna, RajkowskaYong-hak, KimKatarzyna, KotfisYongkang, YangKatarzyna, WiktorskaYongli, ZhangKatarzyna, Otulak-KoziełYonglong, WeiKatarzyna, TonskaYongming, BaoKatarzyna, Komosinska-VassevYongsheng, TaoKaterina, DedkovaYoshihiro, ShidojiKatherine D., McreynoldsYoshihisa, HirakawaKatherine L., AkingbadeYoshikazu, KitanoKathleen R., MarkanYoshiki, NakashimaKatja, SeipelYoshiki, KatayamaKatrien, De BockYoshimichi, HagiwaraKavitha, PathakotiYoshitaka, NagaiKazuharu, NomuraYou-Jin, JeonKazuhide, OkudaYoung-Chul, LeeKazuhiko, KotaniYoung-Gyu, KoKazuki, IdeYraima, CordeiroKazushi, InoueYu, ChenKazuyuki, TakaiYu, ZhangKeiichiro, MatobaYu, AnKeiji, KubaYu, ToyodaKeith, MartinYu min, LeungKeith R., WeningerYuan, WangKelly Aparecida, Geraldo YoneyamaYubin, LiKen, AsadaYu-Chih, ChenKen L., MonsonYu-Chung, ChiangKendall, BylerYuemao, ShenKenichi, YoshikawaYuen, ClementKenji, KomatsuYuerong, LiangKenji, IkeharaYuh, BabaKenji, TanabeYuichi, TsuchiyaKenji, SaitohYukako, HiharaKenji, SobueYukari, OdaKenneth, WitwerYukiko, MatsuoKenneth, D'SouzaYukiko, YoshidaKenneth J., OlejarYuliya, KlymenkoKensuke, KumeYunfei, LiKerry, MillsYung-Chih, ChenKetav, KulkarniYung-Hyun, ChoiKeun-Yeong, JeongYunlong, YuKevin, DaviesYun-Wen, ZhengKevin, SlepYusuke, KatoKhoon, LimYuta, KudoKhosrow Sayed, TayebatiYutaka, KurodaKhurshid, AhmadYutaka, KishidaKi Sung, KangYves, HsiehKilho, EomZdenek, WimmerKim, KeelingZdzisława, Romanowska-DudaKim, KultimaZeba, UsmaniKim A., SharpZhang, YuKimberly, MartinodZhanying, ZhangKirill, GolokhvastZhe, WangKirubakaran, PalaniZhe, QiangKizito, AdejumoZhen, GuKlaus, ReuterZheng, FuKlaus, DornmairZheng, WangKlebson, SantosZhengyu, CaoKo, FujimoriZhentian, LeiKohei, TorikaiZhe-Shan, QuanKoichi, KatoZhibin, LiangKoichi, FurukawaZhifu, SunKoichi, HonkeZhijie, XuKok Lun, PangZhijun, WangKomal, KalaniZhiping, LiuKonda Reddy, KarnatiZhiqun, XieKonrad, MeisterZhixia, LiuKonstantin, AndreevZhonghu, LiKonstantin, VolchoZhonghua, SunKonstantinos, GardikisZhong-Ru, XieKonstantinos, PapoutsisZhuo, WangKonstantinos, PapadimitriouZiv, RadisavljevicKoraljka, Gall TroseljZoe, WaltersKostas, KasiotisZoltán, GáspáriKosuke, SaitoZongmin, ZhaoKosuke, NishiZorita, DiaconeasaKrishna Mohan, SepuruZoubir, DjeradaKrishnendu, KhanZsofia, BanfalviKristen M., WardZsolt, SzegletesKristina, KantminienėZuanning, YuanKristina, NyströmZulfiqar, AhmadKrystyna, MakowskaZuoti, XieKrzysztof, MarciniecZuzana, Macek Jílková

